# The changes in fear of childbirth in pregnancy during and before the COVID-19 pandemic

**DOI:** 10.1038/s41598-024-61307-9

**Published:** 2024-05-14

**Authors:** Cenk Soysal, Özlem Ulaş, Mehmet Murat Işıkalan, İsmail Bıyık, Yasemin Taşçı, Nadi Keskin

**Affiliations:** 1 Department of Obstetrics and Gynecology, Kütahya Health Sciences University, Kutahya, Turkey; 2 Department of Obstetrics and Gynecology, Necmettin Erbakan University, Konya, Turkey; 3 Department of Obstetrics and Gynecology, Faculty of Medicine, Kutahya Health Sciences University, Evliya Çelebi Campus on Tavşanlı Road 10. km, Kutahya, 43020 Turkey

**Keywords:** COVID-19, Education status, Fear of childbirth, Wijma delivery expectancy/experience questionnaire, Psychology, Patient education

## Abstract

We aimed to investigate how factors such as age, education level, planned delivery method and fear of childbirth were affected in pregnant women before and during the pandemic. This cross-sectional study compared a pre-pandemic pregnant group (July 2019 and December 2019) and a pandemic group (November 2020 and May 2021) of patients at Kütahya Health Sciences University Evliya Çelebi Training and Research Hospital. A total of 696 pregnant women in their second trimester were included in the study. All of them were literate and voluntarily agreed to participate in the study. Data were collected with the Wijma delivery expectancy/experience questionnaire (WDEQ-A), and the outpatient doctor asked the questions face-to-face. The mean age of the pregnant women participating in the study was 31.6 ± 6.8 years. While the total Wijma score was 62.1 ± 25.1 in the pre-pandemic group, it was 61.3 ± 26.4 in the pandemic group, and there was no significant difference between the two groups (p = 0.738). Upon analyzing the fear of childbirth among groups based on education level, no statistically significant differences were observed between the pre-pandemic and pandemic periods within any of the groups. While 25.7% (n = 179) of all participants had a normal fear of childbirth, 22% (n = 153) had a mild fear of childbirth, 27% (n = 188) had a moderate fear of childbirth, and 25.3% (n = 176) had a severe fear of childbirth (Wijma score of 85 and above). When the pre-pandemic and the pandemic period were compared, the fear of childbirth was unchanged in pregnant women at all education levels (p = 0.079, p = 0.957, p = 0.626, p = 0.539, p = 0.202). When comparing fear of childbirth before and after the pandemic, it was found that patients with a high school education level have a significantly higher fear of childbirth. To alleviate the fear of childbirth in pregnant women who have completed high school, training or psychosocial support interventions may be prioritized.

## Introduction

Coronaviruses are a family of infectious viruses that cause severe respiratory diseases (MERS-CoV and SARS-CoV). It is known that this new type of coronavirus infection (COVID-19) has a zoonotic origin and is transmitted from person to person. This disease, which first appeared in China (Hubei–Wuhan) and spread worldwide within a short time, has negatively affected the lives of everyone both economically and psychologically^1^.

Studies are still ongoing to understand the effects of COVID-19 infection during pregnancy. There is currently no evidence that pregnant women are at higher risk of contracting COVID-19 disease than the general population^2,3^. Recent evidence indicates that pregnant women are at higher risk for severe illness and adverse outcomes from COVID-19 infection. A large-scale study conducted by the Centers for Disease Control and Prevention (CDC) in the United States has shown that pregnant women with COVID-19 are more likely to require intensive care unit admission, mechanical ventilation, and extracorporeal membrane oxygenation (ECMO) support compared to non-pregnant women with COVID-19^4^. In addition, several studies have reported an increased risk of preterm birth, fetal distress, neonatal intensive care unit admission, and stillbirth in pregnancies complicated by COVID-19^5^. These increased risks are likely related to the changes in the immune system of pregnant women. As a result, it is crucial for pregnant women to take precautions to protect themselves during the COVID-19 pandemic and to seek medical attention immediately if they experience possible symptoms, such as fever, cough, or breathing difficulty. However, due to the changes in the immune system of pregnant women, it is important for them to take precautions to protect themselves during COVID-19 and to apply to healthcare professionals immediately when possible symptoms (such as fever, cough, or breathing difficulty) occur^6^.

In studies to examine the clinical features of COVID-19 infection and the potential for congenital infection, SARS CoV-2 was tested in amniotic fluid, cord blood, newborn throat swab, and breast milk samples, and all samples were found to be negative for the virus^7,8^. However, pregnant women may be concerned about the possibility of transmission of the infection to the fetus and may be more prone to anxiety^9^. In the literature, it has been reported that pregnant women evaluated after the declaration of the COVID-19 pandemic showed significantly higher depression symptoms than those evaluated before the pandemic^10^. Pregnancy stress management is a crucial concept that aids expectant mothers in maintaining their physical and emotional well-being. Stress experienced during pregnancy can be a detrimental factor for both the mother and the baby^11^. Therefore, successfully managing stress is essential for completing the pregnancy process healthily.

The pandemic period is already a stressful time for people^12^. During pregnancy, uncertainties and health concerns caused by the pandemic can further elevate stress levels for mothers. The pandemic brings additional factors that complicate the pregnancy process, such as limitations in access to social support systems, overcrowding in hospitals, and concerns about the risk of infection^13^. This situation underscores the importance of stress management during pregnancy and highlights the need for expectant mothers to adopt effective coping strategies during the pandemic.

When reviewing the literature, it can be observed that the fear of childbirth is a condition present in all nulliparous or multiparous pregnant women^14^. Anxiety can be a concern that affects not only the mental health of the mother but also the birth and postpartum periods^15^. Studies investigating the association between fear of childbirth and parity have revealed that primiparous pregnant women experience a greater fear of labor compared to multiparous ones^16^.

In this study, we aimed to investigate the effect of the COVID-19 pandemic on the fear of childbirth in terms of the age, education level and parity of the pregnant women who gave birth before and during the pandemic. This study will contribute to the relevant literature and may be a guide to the importance of mental health assessment of pregnant women in adverse situations like pandemics.

## Materials and methods

The COVID-19 pandemic group of this cross-sectional study consisted of pregnant women in their second and third trimesters, all of whom were literate and applied to Kütahya Health Sciences University Evliya Çelebi Training and Research Hospital between November 2020 and May 2021. This cross-sectional cohort study compared pregnant women who were enrolled during the first and second waves of the COVID-19 pandemic (participants) with pregnant women who were enrolled prior to the pandemic (reference group). The fear of childbirth group in the pre-pandemic group was formed with the data in our study, which were collected in 2019^17^. Ethical approval was received from the Ethics Committee of Kutahya Health Sciences University on 21.10.2020 with decision number 2020/15-04. The study was carried out according to principles of the Declaration of Helsinki.

The survey comprised two parts: a demographic questionnaire and a validated self-report questionnaire. The demographic questionnaire included variables such as maternal age, parity, marital status, history of previous treatment for psychological issues, and residency in a COVID-19 risk area. During patient selection, those having fetal anomalies, or complications (gestational diabetes, preeclampsia, macrosomic fetus or maternal chronic diseases), as well as those diagnosed with psychiatric illness, a previous history of abortion and divorced or separated from their spouses were excluded from the study. Questionnaires were conducted with the patients by the outpatient clinic doctor face to face.

For the purpose of this study, we also collected information on gestational week, which was then recorded along with the demographic data. A 33-question Wijma delivery expectancy/experience questionnaire (WDEQ-A) questionnaire was completed. Questions 2, 3, 6, 7, 8, 11, 12, 15, 19, 20, 24, 25, 27, and 31 in the questionnaire were calculated by inverting them in accordance with the purpose of questionnaire scoring. The WIJMA is a widely used questionnaire that has been shown to have high internal consistency. We used the Turkish version of the WIJMA in our study, which has been validated in a previous study^18^. Regarding the participants and recruitment process, we recruited pregnant women in their third trimester from two hospitals in Istanbul, Turkey. Previous studies have suggested that pregnant women with a score of 85 or above on the Wijma delivery expectancy/experience questionnaire (WIJMA) fear of childbirth scale are experiencing severe fear of childbirth^19^.

### Power analysis

The G*Power 3.1 statistical analysis program was used to calculate the sample size. The error probability, effect size f value, power of the study and total sample size were 0.05, 0.10, 0.8 and 429, respectively.

### Statistical analysis

All data collected for statistical analysis were analyzed with the Statistical Package for the Social Sciences, version 25, SPSS Inc., Chicago, IL (SPSS). Whether the data showed normal distribution for each group was evaluated with the Kolmogorov Smirnov test. Normally distributed groups were compared with Student’s T test, while the Mann Whitney U test was used for data that did not fit the distribution. A one-way analysis of variance (ANOVA test) for multiple comparisons or the Kruskal Wallis test was used for nonparametric multiple analysis. Differences between the groups were evaluated using the Tukey test or Tamhane’s T2 test. Values with a statistical significance p value below 0.05 were defined as significant. Reliability analysis was performed, and Cronbach’s Alpha value was found to be 0.931.

### Informed consent

Consent to participate and for publication Health authority permission, written informed consent from all participants as well as hospital managers’ approval, is available for this study.

## Results

The age of all the pregnant women in the study ranged between 18 and 43, and the mean age was 31.6 ± 6.8 years. The mean gestational age was 33.9 ± 3.3 weeks, while the mean WDEQ-A score of all participants, both the pre-pandemic and pandemic groups, was 61.8 ± 25.6. The demographic characteristics of the participants according to the groups are shown in Table 1.

**Table 1:** Demographic characteristics of the groups.

	Pre-pandemic (n = 444)	Pandemic (n = 252)	*p* value
Age (year)*	32 ± 7	32 ± 7	0.989
Parity**	2 (0–4)	1 (0–4)	0.241
Gestational age (week)*	35 ± 3	32 ± 4	**p < 0.001**

*Mean ± standart deviation.

**Median (min–max).

Significant p values are represented in bold.

In the pre-pandemic period, the distribution of multiparous and nulliparous patients was 36% nulliparous and 64% multiparous. However, during the pandemic period, the patient group demonstrated a slightly different distribution with 33% nulliparous and 67% multiparous.

In the evaluation of fear of childbirth according to age, no significant difference was observed in terms of WDEQ-A scores between the pre-pandemic and pandemic periods (p = 0.951, p = 0.950, p = 0.885, p = 651, respectively). However, for all participants, the fear of childbirth was observed to be significantly increased for those aged 30 and above compared to those below the age of 30 (p < 0.001) (Table 2).

**Table 2:** Fear of childbirth by age among groups.

	Pre-pandemic (n = 444)	Pandemic (n = 252)	*p* value
18–24 years	56 ± 21	55 ± 21	0.951
25–29 years	58 ± 24	58 ± 25	0.950
30–34 years	67 ± 27	67 ± 27	0.885
35 years and older	65 ± 26	64 ± 29	0.651

The values has given mean ± standard deviation.

There was no statistically significant difference in terms of WDEQ-A scores when comparing the education levels of the pregnant women (elementary, middle school, high school, collage, master and above) in the pre-pandemic and pandemic groups (p = 0.079, p = 0.957, p = 0.626, p = 0.539, p = 0.202 respectively). However, it was observed that WDEQ-A scores for fear of childbirth increased significantly as the education level of the groups increased (p < 0.001, p < 0.001, respectively) (Table 3).

**Table 3:** Fear of childbirth by education level among groups.

	Pre-pandemic (n = 444)	Pandemic (n = 252)	*p* value
Elementary**	29 (22–35)	38 (23–101)	0.079
Middle school**	42 (30–78)	42 (22–79)	0.957
High school**	72 (60–89)	69 (23–107)	0.626
College**	89 (76–109)	91 (24–109)	0.539
Master and above**	91 (71–111)	95 (25–111)	0.202
Total Wijma score*	62.1 ± 25.1	61.3 ± 26.4	0.738

*Mean ± standard deviation.

**Median (min–max).

When severe fear of childbirth according to education levels was examined, it was found that this rate increased from 0.04% in the pre-pandemic period to 17% in the pandemic period for high school graduates (p = 0.003). In other education levels, it was observed that the rates of severe fear of childbirth did not change (p = 0.162, p = 0.932, p = 1.000) (Table 4).

**Table 4:** Severe fear of childbirth by education level.

	Pre-pandemic (n = 444)	Pandemic (n = 252)	*p* value
Elementary	0/87 (0%)	2/59 (0.03%)	0.162
Middle school	0/108 (0%)	0/46 (0%)	*
High school	4/110 (0.04%)	15/88 (17%)	**0.003**
College	50/70 (71%)	26/36 (72%)	0.932
Master and above	54/69 (78%)	18/23 (78%)	1.000
Total	108/444 (24%)	61/252(24%)	1.000

*Statistical analysis cannot be performed due to lack of observations.

Significant p value are represented in bold.

While 25.7% (n = 179) of all participants had a normal fear of childbirth (WDEQ-A score of 37 or less), 22% (n = 153) had a mild level (WDEQ-A score of 38–65), 27% (n = 188) had a moderate level (WDEQ-A score of 66–85) and 25.3% (n = 176) had a severe level of fear of childbirth (WDEQ-A score of 85 or above)^20^.

## Discussion

Pregnancy and childbirth are physiological processes that affect women’s lives. Although pregnant women may express it in different ways, many of them face anxiety and fear of childbirth^21^. Lifestyle changes due to thevCOVID-19 pandemic place a further psychological burden on pregnant women, so they experience more anxiety and uncertainty during pregnancy than ever before^22^. In our study, it was observed that the fear of childbirth in pregnant women did not change with education levels before and during the COVID-19 pandemic. It was observed that fear of childbirth did not change with age groups and parity between the pre-pandemic and the pandemic period.

Pregnant women who received the SARS-CoV-2 vaccine had a lower risk of severe maternal morbidity and mortality. On the other hand, women who had a caesarean section, contracted SARS-CoV-2 during their third trimester, or had comorbidities such as asthma, chronic hypertension, or obesity had an increased risk^23^.

The restrictions in place during the pandemic and the uncertainty about when they would be able to return to normal living conditions create stress in pregnant women^24^. It must be emphasized that stress management is an important issue during pregnancy, as stress factors that cannot be eliminated during pregnancy can cause negative pregnancy outcomes^25^. For this reason, health professionals should question the psychological status of patients related to anxieties during pregnancy follow-up and ensure that they receive psychiatric help when necessary. The study we conducted before the pandemic showed that as the education level increases, the fear of childbirth scores, as measured by the WDEQ-A score, also increase^17^. In our current study conducted with the same scale, fear of childbirth increased with education level, but according to the study conducted before the pandemic period, it was seen that the fear of childbirth scores did not increase statistically at all education levels during the pandemic period. In studies conducted before the pandemic in Turkey, the mean fear of childbirth score was lower than during the pandemic period^17,26,27^.

While the COVID-19 pandemic increases the frequency of anxiety, stress, and depression worldwide, it can cause additional fear and anxiety in pregnant women due to concern for their baby^28^. The general reasons for this are inadequate health systems, lockdowns during the pandemic, cancellation of hospital appointments, the possibility of contamination to the newborn during pregnancy, birth or after birth, and the mother’s fear of being separated from her baby in this period. Similarly, in the studies conducted during the pandemic period in the literature, the opinion that the fear of childbirth is increasing is dominant^9,21,28,29^. A study in Germany, in which 1364 pregnant women participated, found that the Pandemic-Related Pregnancy Scale (PREPS) scores, which they used as a fear of childbirth scale, increased during the pandemic period^30^. Saccone et al.’s study, including 100 pregnant women during the COVID-19 pandemic, reported that nearly half of the pregnant women had serious psychological effects and high anxiety scores^31^. Considering the discussions in the literature, it is expected that the fear of childbirth and anxiety will increase in pregnant women during the COVID-19 pandemic. Our study contradicts the literature in this aspect. The reason for this may be that the studies could not report an increase compared to the pre-pandemic period since they only cover the pandemic period. In our study, no significant difference was found when the pre-pandemic period and the fear of childbirth during the pandemic period were compared. To the best of our knowledge, our study is the first to compare the pandemic period with the pre-pandemic period regarding fear of childbirth.

In a study conducted in Thailand on uncomplicated singleton pregnancies, while the fear of labor was 51.9 ± 14.3, it was lower than our study^32^. The prevalence of a severe fear of childbirth (WDEQ-A score ≥ 85) reported in the publications was 4.5% in the Irish study^33^, 21% in the Turkey meta-analysis^34^, 19.6% in the Iranian study^35^ and 20% in the two Swedish studies^36,37^, whereas it was 24% in our study. An overall look at studies reveals that pregnant women in Turkey have a higher fear of birth than those in other countries. Since studies on fear of childbirth are mostly conducted in Europe and Scandinavian countries, the fear of childbirth may have been found to be higher in our country due to socio-economic differences.

Another important aspect of our study is that as the level of education increases so does fear of childbirth. WDEQ-A scores increase significantly both in the pre-pandemic and the pandemic period. In the studies conducted in Hungary, Denmark, and Finland, the education level was divided into two groups as primary and post-primary education, and the difference between them was examined. In all three studies, it was found that the level of fear of childbirth increased as the level of education decreased^38–40^. The relationship of our research with the level of education contradicts the literature. The reason for this is the effect of social media during the pandemic period in our country. Since the rate of internet access and exposure to visual and written documents is higher in educated pregnant women, they are more knowledgeable about possible birth complications, body deformation and traumatic.

The main limitation of our study is that a comparison of the fear of childbirth cannot be made on the same patients since it is very unlikely that the same woman will give birth in both pre-pandemic and pandemic periods. The strength of the study is that it is the first study in the literature to compare the fear of childbirth before and during the pandemic.

## Conclusion

During the pandemic period, the fear of childbirth is affected by educational status, but these conditions can vary according to the society. By predetermining the conditions that increase the fear of childbirth and providing supportive outpatient clinic services to pregnant women during the pandemic period, the fear of childbirth can be reduced. Doctors can explain that, based on our current knowledge, the possibility of the baby being infected by COVID-19 is minimal. This may also be beneficial in reducing the increasing requests for cesarean deliveries, which may also be affected by maternal anxiety.

## Data Availability

The datasets used and/or analysed during the current study available from the corresponding author on reasonable request.
